# P-911. Antifungal Treatment Patterns, Healthcare Utilization, Costs and Mortality in Coccidioidal Meningitis Patients: A Descriptive Analysis of United States Nationwide Claims Database

**DOI:** 10.1093/ofid/ofae631.1102

**Published:** 2025-01-29

**Authors:** Fariba Donovan, Mark Bresnik, Lia Pizzicato, Ruthwik Anupindi, Mitchell DeKoven, Belinda Lovelace, Craig I Coleman

**Affiliations:** University of Arizona, Valley Fever Center for Excellence, Tucson, Arizona; F2G, Ltd., Princeton, New Jersey; IQVIA, Philadelphia, Pennsylvania; IQVIA, Philadelphia, Pennsylvania; IQVIA, Philadelphia, Pennsylvania; F2G, Inc., Princeton, New Jersey; University of Connecticut, Storrs, Connecticut

## Abstract

**Background:**

Coccidioidal meningitis (CM) patients require lifelong antifungal therapy (AFT) to reduce morbidity and mortality. This study sought to identify CM patients and characterize their AFT patterns, healthcare utilization, costs, and mortality.

**Table 1.**
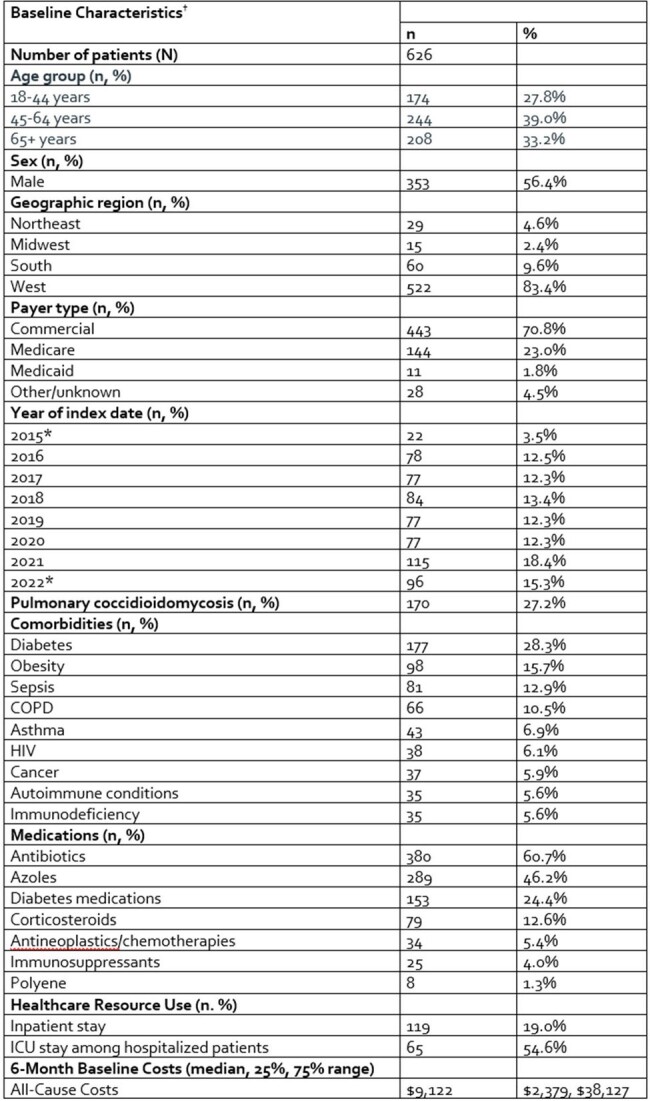
Baseline Characteristics of Patients with Coccidioidal Meningitis *Partial years data utilized †Baseline period was defined as the 6 months prior to, but not including the date of coccidioidal meningitis diagnosis (index date)

**Methods:**

A retrospective study was performed in the United States (US) using data from IQVIA’s New Data Warehouse (hospital, outpatient and prescription claims) for 2015-2022. We identified CM patients at time of diagnosis (index date). AFT use patterns were captured for up to 4 lines of therapy per patient. Subsequent lines began upon discontinuation and restart, switch or modification of first line AFT. Healthcare utilization and treatment costs were depicted as per person per year (PPPY). Results were reported as frequencies or medians (25%, 75% ranges).

**Figure 1.**
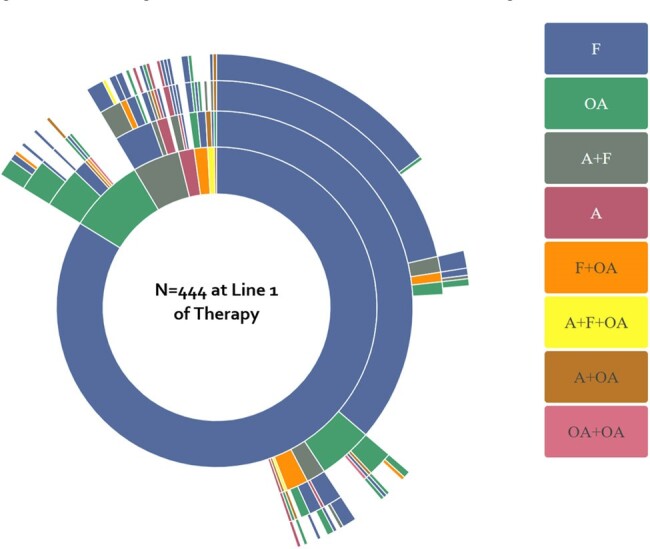
Antifungal Treatment Patterns in Patients Diagnosed with Coccidioidal Meningitis A=amphotericin B; F=fluconazole; OA=other azole

**Results:**

In total, 626 CM patients were identified with a follow up of 23 (9, 50) months. Median age was 57 (42, 68) years, 56.4% were men, 83.4% from the Western US and 70.8% had commercial insurance (**Table 1**). Common comorbidities included diabetes, obesity and pneumonia. In total, 70.9% of patients initiated first line AFT, for which 10 unique treatment courses accounted for 82.0% of patients (**Figure 1**). Azoles were used as first line AFT in 98.4% of patients, with 83.8% receiving fluconazole monotherapy. Polyenes were used in 7.2% of patients as first line AFT, with 1.6% as monotherapy. First line AFT lasted 31 (7, 224) days. Second, third- and fourth-line AFT was observed in 257, 182 and 129 patients, respectively. Azoles continued to be used often over subsequent lines of AFT (96.9-97.7% of patients), with 66.5-77.5% of azole use being fluconazole monotherapy. Polyene use increased (peaking at 13.2% of patients in line 3), often administered with azoles (55.6-79.2%). Patients experienced 0.81 (0.37, 2.28) hospitalizations and 5 (1, 12) outpatient physician office visits PPPY after the index diagnosis encounter. Total all-cause costs were $28,664 ($9,912, $89,460) PPPY. All-cause death occurred in 5.4% of patients (time to death=79 (9, 418) days).

**Conclusion:**

This study demonstrates the complexity of treating CM in routine practice. CM is responsible for a substantial clinical and economic burden in the US.

**Disclosures:**

**Fariba Donovan, MD, PhD**, F2G: Advisor/Consultant|F2G Ltd: Advisor/Consultant|F2G Ltd: Grant/Research Support|F2G Ltd: Honoraria **Mark Bresnik, MD**, F2G Ltd: Employee **Lia Pizzicato, MPH**, F2G Ltd: Grant/Research Support|IQVIA: Employee **Ruthwik Anupindi, PhD**, F2G Ltd: Grant/Research Support|IQVIA: Employee **Mitchell DeKoven, PhD**, F2G Ltd: Grant/Research Support|IQVIA: Employee **Belinda Lovelace, PharmD, MS, MJ**, F2G, Inc.: Employee **Craig I. Coleman, PharmD**, F2G Ltd: Advisor/Consultant|F2G Ltd: Grant/Research Support

